# Leptin-inhibited neurons in the lateral parabrachial nucleus do not alter food intake or glucose balance

**DOI:** 10.1080/19768354.2022.2084159

**Published:** 2022-06-07

**Authors:** Seahyung Park, Kevin W. Williams, Jong-Woo Sohn

**Affiliations:** a Department of Biological Sciences, Korea Advanced Institute of Science and Technology (KAIST), Daejeon, Korea; b Center for Hypothalamic Research, Department of Internal Medicine, University of Texas Southwestern Medical Center, Dallas, TX, USA

**Keywords:** Appetite, glucose, leptin, parabrachial nucleus, chemogenetics

## Abstract

The lateral parabrachial nucleus (LPBN) has been shown to be involved in the suppression of appetite at the pharmacological, optogenetic and chemogenetic levels. However, the signalling that mediates activation of these neurons in physiological conditions has been hindered by difficulties in segregating different cell populations in this region. Using reporter mice, we identify at the electrophysiological level the effects of an anorexic hormone, leptin, on leptin receptor (ObR)-expressing neurons in the LPBN (LPBN^ObR^ neurons). Application of leptin caused inhibition in a subpopulation of LPBN^ObR^ neurons. This effect was mediated by an increased potassium conductance and was also accompanied by a decrease in excitatory synaptic input onto these neurons. However, mimicking the inhibitory effects of leptin on LPBN^ObR^ neurons through chemogenetics led to no changes in feeding or glucose levels, which suggests that leptin action on LPBN^ObR^ neurons may not be sufficient to regulate these metabolic aspects.

## Introduction

Obesity is a growing problem worldwide, which leads to various health complications such as hypertension and diabetes. This inevitably causes huge healthcare costs that impact not only on the lives of patients in terms of health and self-esteem but also indirectly causes problems on normal tax-payers lives through paying for health-care expense. Despite these health, psychological and economic issues, effective ways to combat obesity have yet to be developed. Since obesity occurs through over-eating, one simple but effective solution would be to curb appetite by modulating the brain regions that are involved in producing the feelings of hunger and satiety.

The adipocyte-secreted hormone leptin is known to suppress appetite. Indeed, complete abolishment of *Ob*, the gene encoding for leptin, leads to obese phenotypes (Zhang et al. 1994), which is rescued by exogenous supplementation of leptin (Campfield et al. 1995; Halaas et al. 1995; Pelleymounter et al. 1995; Farooqi et al. 1999). In wildtype mice, administration of leptin has been shown to decrease food intake and weight gain (Halaas et al. 1995). These data support the notion that leptin acts to regulate weight in part by reducing appetite.

Past research has implicated the lateral parabrachial nucleus (LPBN) in the suppression of appetite at the pharmacological (Wu et al. 2009), optogenetic and chemogenetic levels (Carter et al. 2013; Campos et al. 2016). Chronic stimulation of these neurons leads to drastic weight loss and can eventually lead to starvation in mice, suggesting that these neurons play a major role in the suppression of appetite. Previous studies have suggested that the LPBN contains functional leptin receptors (ObR) through c-Fos staining (Elmquist et al. 1997), STAT3 phosphorylation (Hosoi et al. 2002) and mRNA hybridisation (Mercer et al. 1998; Grill et al. 2002). Consistent with their anorexigenic roles, exogenous leptin infused into the LPBN results in a decrease of meal sizes in rats (Alhadeff et al. 2014). Taken together, these studies suggest that ObR-expressing neurons in the LPBN may play a role in regulating appetite.

In this study, we aimed to identify the electrophysiological effects leptin has on LPBN neurons and what effects ObR-expressing neurons in the LPBN have on appetite and glucose regulation.

## Methods

### Animals

Mice were housed at 22–24°C with a 12 h light:12 h dark cycle on normal chow (Teklad, 2018S). ObR-IRES-Cre::tdTomato mice were generated by crossing ObR-IRES-Cre mice (Jax #008320) and tdTomato reporter mice (Jax #007908). Food and water was provided *ad libitum* when experiments were not being conducted. 6- to 8-week-old male homozygous and heterozygous mice were used for surgery. No differences in results were observed between the two genotypes, thus results were pooled together. Allocation of animals to control or experimental groups were randomised. Investigators were not blinded to group allocation. All procedures were conducted according to the Korean Advanced Institute of Science and Technology (KAIST) Guidelines for the Care and Use of Laboratory Animals and were approved by the Institutional Animal Care and Use Committee (Protocol No. KA2021-126).

### Stereotaxic injections

Mice were anaesthetised under isoflurane and had their heads fixed in a stereotaxic frame. Lidocaine (2% wt/vol) was applied topically for pre-emptive analgesia. The skull was drilled and a 33 g blunt NanoFil needle (World Precision Instruments, NF33BL-2) was lowered to the target region (LPBN: A-P 5.15 mm, M-L 1.25 mm, D-V 3.3 mm). For chemogenetic experiments, 0.2–0.3 μl of AAV2-hSyn-DIO-hM4Di-mCherry (University of North Carolina Vector Core; 3.7 × 10^12^ molecules/μl) was injected into the LPBN of ObR-IRES-Cre mice using a Hamilton syringe at a rate of 0.2 μl/min. 7–10 min after injection of the virus, the needle was then removed slowly. Mice were kept on a heating pad and watched closely until regaining movement. Mice were handled for 1 week before starting behavioural experiments. At least 3 weeks of recovery was given before starting behavioural experiments.

### Behavioural experiments

For fast-refeeding experiments, mice were housed in single cages for at least 1 week before conducting assays. The day before the assay, mice body weights were measured, food was cleared and bedding was replaced. Stock CNO was dissolved in DPBS at 8.75 mM. Stock CNO was diluted in sterile saline and administered intraperitoneally at 1 mg/kg, whereas DPBS was administered by diluting in sterile saline at a ratio consistent with CNO injections. In the text, saline injections refer to DPBS diluted in sterile saline. Injections were given 18 h after fasting, at 10:00 on the day of the assay. Food was then reintroduced (either normal chow, sodium deficient diet or control diet) and then food intake and body weight were measured at 1, 2, 4 and 6 h after intraperitoneal (i.p.) injections.

For glucose-tolerance tests, mice were housed in single cages for at least 1 week before conducting assays. The day before the assay, mice body weights were measured, food was cleared and bedding was replaced. Stock CNO was dissolved in DPBS at 8.75 mM. Stock CNO was diluted in sterile saline and administered intraperitoneally at 1 mg/kg, whereas DPBS was administered by diluting in sterile saline at a ratio consistent with CNO injections. In the text, saline injections refer to DPBS diluted in sterile saline. Injections were given 15 h and 15 min after fasting, at 8:15 on the day of the assay. A baseline blood glucose measurement was taken before administering a bolus of glucose solution (1.25 mg/kg, dissolved in saline) intraperitoneally. Blood glucose was then measured in intervals.

For insulin-tolerance tests, mice were housed in single cages for at least 1 week before conducting assays. On the day of the assay, mice body weights were measured, food was cleared and bedding was replaced. Stock CNO was dissolved in DPBS at 8.75 mM. Stock CNO was diluted in sterile saline and administered intraperitoneally at 1 mg/kg, whereas DPBS was administered by diluting in sterile saline at a ratio consistent with CNO injections. In the text, saline injections refer to DPBS diluted in sterile saline. Injections were given 3 h and 15 min after fasting, at 13:15 on the day of the assay. A baseline blood glucose measurement was taken before administering a bolus of insulin solution (1.5 mU/g), intraperitoneally. Blood glucose was then measured in intervals.

### Histology

After the completion of behavioural experiments, mice were perfused with saline and 4% PFA. Brains were extracted and post-fixed for 24 h at 4°C in 4% PFA. 50 μm sections were then made on a vibratome (Leica VT1200S) and mounted with Vectashield mounting medium with DAPI, Hard Set (Vector, H-1500). Mice were excluded from analysis if vector expression was absent, incomplete or outside the area of interest.

### Electrophysiology

Whole-cell patch-clamp recordings from ObR-expressing neurons were made from the LPBN of 3- to 6-week old male and female mice, as described previously (Park et al. 2020). Briefly, mice were anaesthetized with isoflurane and transcardially perfused with a cutting solution (220 mM sucrose, 26 mM NaHCO_3_, 2.5 mM KCl, 1 mM NaH_2_PO_4_, 5 mM MgCl_2_, 1 mM CaCl_2_, 10 mM glucose, pH 7.3–7.35). The mice were then decapitated, and the entire brain was removed and immediately submerged in ice cold, carbogen-saturated cutting solution. 300 μm coronal sections were cut from the LPBN with a Leica VT1200S Vibratome and then incubated in oxygenated storage solution (123 mM NaCl, 26 mM, NaHCO_3_, 2.8 mM KCl, 1.25 mM NaH_2_PO_4_, 1.2 mM, MgSO_4_, 2.5 mM CaCl_2_, 10 mM glucose pH 7.3–7.35) at 34°C for at least 1 h before recording. Slices were transferred to the recording chamber and allowed to equilibrate for 10 min before recording. Recordings were made in the presence of a recording solution (126 mM NaCl, 26 mM, NaHCO_3_, 2.8 mM KCl, 1.25 mM NaH_2_PO_4_, 1.2 mM, MgSO_4_, 2.5 mM CaCl_2_, 5 mM glucose pH 7.3–7.35). The pipette solution for current-clamp whole-cell recording was modified to include an intracellular dye (Alexa Fluor 488): 120 mM K-gluconate, 10 mM KCl, 10 mM HEPES, 5 mM EGTA, 1 mM CaCl_2_, 1 mM MgCl_2_, 2 mM MgATP (pH 7.29). The pipette solution for voltage-clamp whole-cell recording was modified to include an intracellular dye (Alexa Fluor 488): 120 mM Cs-gluconate, 10 mM KCl, 10 mM HEPES, 5 mM EGTA, 1 mM CaCl_2_, 1 mM MgCl_2_, 2 mM MgATP (pH 7.29). Miniature EPSCs and IPSCs were recorded in the presence of TTX (500 nM) at holding voltages of −60 mV and −10 mV respectively. Recordings with access resistances above 25 MΩ and/or had changed more than 20% at the end of the recording were discarded. Epifluorescence was briefly used to target fluorescent cells, at which time the light source was switched to infrared differential interference contrast imaging to obtain the whole-cell recording (Nikon Eclipse FN-S2N equipped with a fixed stage and a QImaging optiMOS sCMOS camera). Electrophysiological signals were recorded using an Axopatch 700B amplifier (Molecular Devices), low-pass filtered at 2–5 kHz, and analyzed offline on a PC with pCLAMP programmes (Molecular Devices). Recording electrodes had resistances of 2–6 MΩ when filled with internal solutions. Input resistance was assessed by measuring voltage deflection at the end of the response to hyperpolarizing rectangular current pulse steps (500 ms, from −5 to −25 pA, −10 to −50 pA or −20 to −100 pA). Membrane potential values were not compensated to account for junction potential (−8 mV).

### Drugs

Leptin was acquired from A. F. Parlow through the National Hormone and Peptide Program. TTX was acquired from Tocris. Lidocaine and CNO were acquired from Sigma. Stock solutions of TTX, were made by dissolution in DW. Stock solutions of CNO were made in DPBS (Sigma, D8537).

### Statistics

All statistics were done using Prism 6.01 (GraphPad) software. For electrophysiology experiments, nonparametric statistical tests (two-tailed Wilcoxon signed rank test) were used. For electrophysiology experiments, n represents the final number of recorded cells. All chemogenetic experiments were analysed using two-way repeated measures ANOVA, matched by time and treatment. N represents the final number of validated healthy animals. Sample sizes were not predetermined, but matched to similar literature.

## Results

### Electrophysiological effects of ObR activation in the LPBN

**Figure 1.**
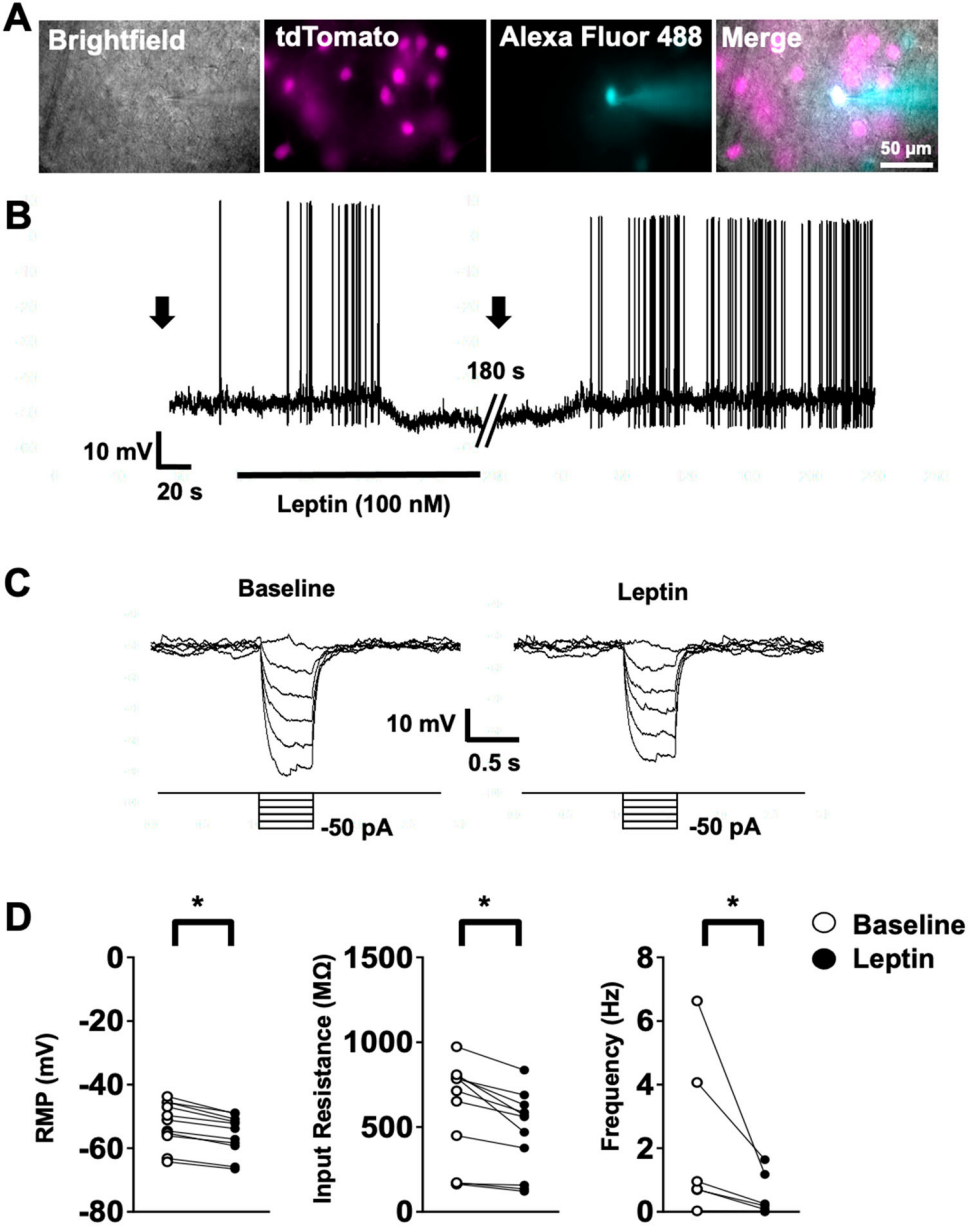
Leptin inhibits LPBN^ObR^ neurons. (A) Brightfield illumination, tdTomato, Alexa Fluor 48 and merged pictures of a patched cell (from left to right). (B) Leptin application hyperpolarises a subset of LPBN^ObR^ neurons. Continuous recordings were interrupted to apply current step pulses as indicated (arrows). (C) Hyperpolarisation by leptin is accompanied with a decreased input resistance, measured by deflection of membrane in response to hyperpolarising current steps. (D) Responsive cells hyperpolarise and have a decreased input resistance. Spontaneously firing cells show decreased action potential frequency. *P < 0.05 (Wilcoxon rank-sum test). Responsiveness was defined as a stabilised change > 2 mV that followed an exponential time course, associated with a change in input resistance in response to leptin application.

To investigate the electrophysiological effects of ObR stimulation in the LPBN, we generated ObR-IRES-Cre::tdTomato reporter mice (see methods) to label all ObR-expressing cells with tdTomato. Whole-cell patch clamp recordings were then taken under fluorescent guidance (Figure 1A). We found that leptin hyperpolarised a subset of tdTomato-labelled, ObR-expressing LPBN (LPBN^ObR^) neurons by −3.4 ± 0.4 mV (*n* = 11), where the action potential frequency was also decreased (Figures 1B and D). The hyperpolarising effect was accompanied by a decrease in input resistance **(**from 587.7 ± 89.8 MΩ to 466.2 ± 72.6 MΩ, *n* = 11, Figures 1C and D), with a reversal potential of −86.1 ± 3.8 mV (*n* = 11), suggesting an increased potassium conductance. Together, these results suggest that leptin acts to hyperpolarise LPBN^ObR^ neurons by opening a putative potassium conductance.

**Figure 2.**
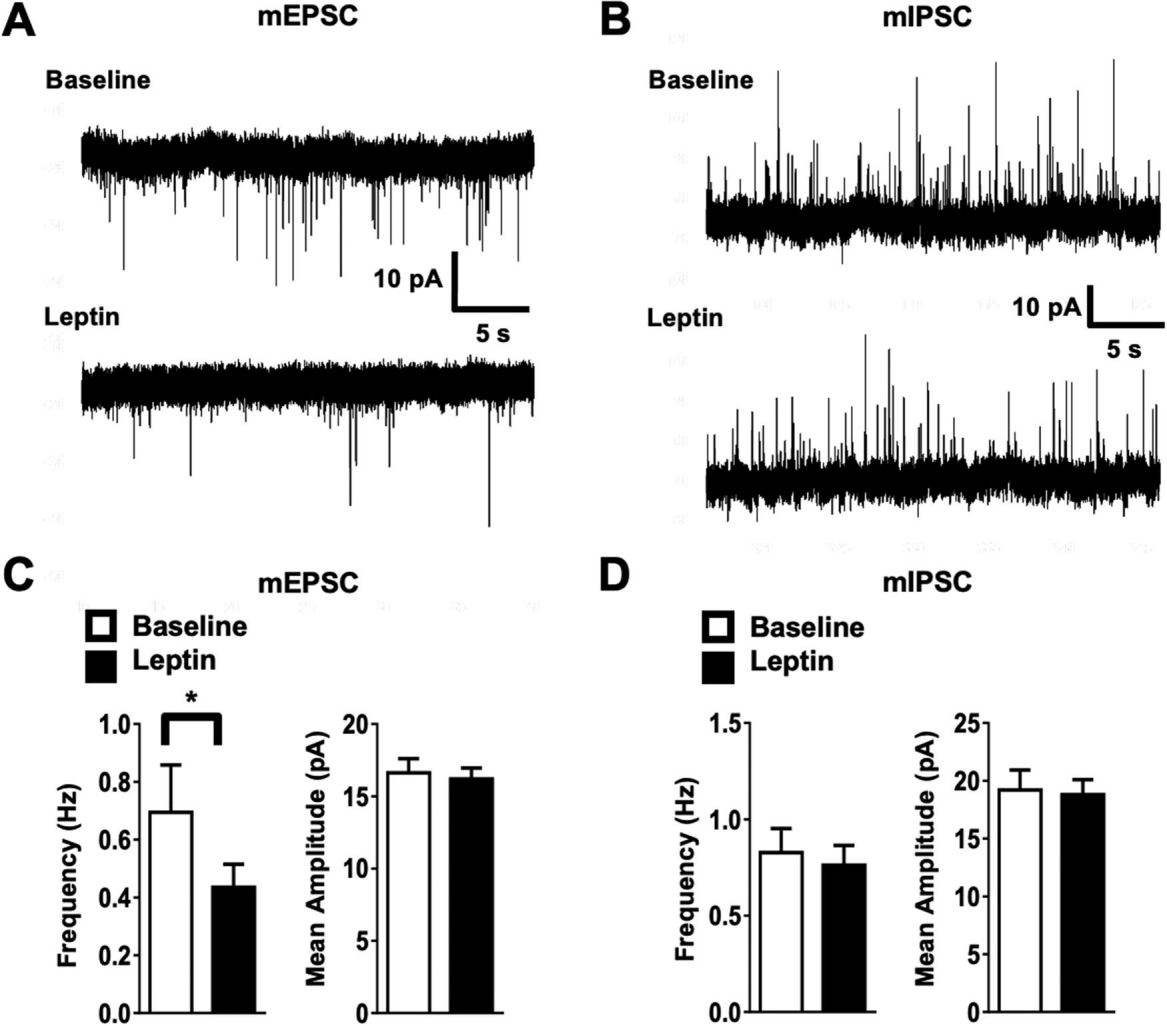
Leptin results in net inhibitory synaptic drive into the LPBN.(A) Leptin decreases frequency, but not amplitude, of mEPSCs in LPBN^ObR^ neurons. (B) Leptin minimally changes frequency or amplitude of mIPSCs. (C) Summary of acute leptin effects on mEPSC (*n* = 11). (D) Summary of acute leptin effects on mIPSC (*n* = 18). *P < 0.05 (Wilcoxon rank-sum test).

We also investigated the synaptic effects of leptin on LPBN^ObR^ neurons by measuring miniature excitatory postsynaptic currents (mEPSCs) and miniature inhibitory postsynaptic currents (mIPSCs). We found that mEPSC frequency decreased without changing amplitude (Figures 2A and C), whereas mIPSCs were unaffected by leptin treatment (Figures 2B and D). Together, these results all suggest that leptin collectively acts to inhibit the activity of LPBN^ObR^ neurons at the cellular and network level.

### Metabolic effects of mimicking ObR activation in the LPBN

**Figure 3.**
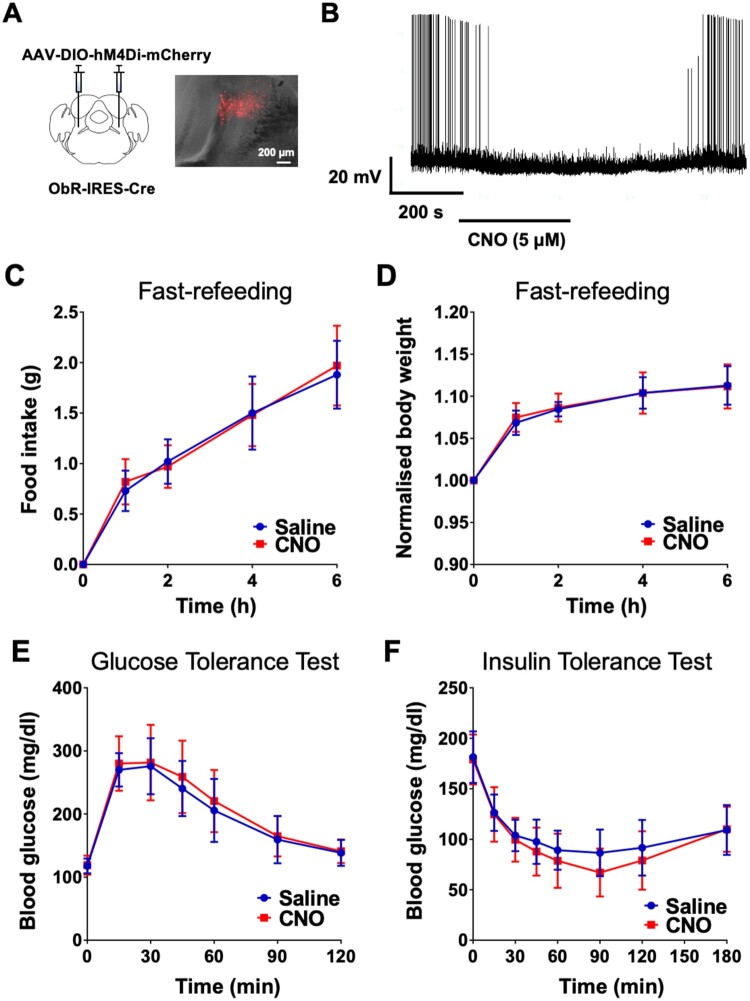
Chemogenetic inhibition of LPBN^ObR^ neurons does not affect feeding or glucose regulation (A) Bilateral injection of an inhibitory DREADD construct (AAV-DIO-hM4Di-mCherry) into the LPBN of ObR-IRES-Cre mice. (B) Confirmation of hM4Di done through patch clamping. Application of CNO reversibly hyperpolarises neurons and reduces action potential frequency. (C) CNO (1 mg/kg) does not affect food intake during a fast-refeeding assay. (D) CNO (1 mg/kg) does not affect body weight during a fast-refeeding assay. Body weight normalised to the beginning of the assay. (E) CNO (1 mg/kg) does not affect blood glucose during a glucose-tolerance test. (F) CNO (1 mg/kg) does not affect blood glucose during an insulin-tolerance test. *N* = 10 mice.

Given the inhibitory effects of leptin on LPBN^ObR^ neurons, we characterised the metabolic role of leptin action on LPBN^ObR^ neurons. To reproduce leptin effects *in vivo*, we bilaterally injected AAV-DIO-hM4Di-mCherry into the LPBN of ObR-IRES-Cre mice (LPBN^ObR-hM4di^ mice) (Figure 3A). We validated this chemogenetic construct *in situ* with whole-cell patch clamp recordings, which showed that application of CNO (5 μM) caused a reversible inhibition of LPBN^ObR^ neurons (−6.1 ± 1.2 mV, *n* = 5 cells) (Figure 3B).

We then tested the effects of LPBN^ObR^ neuronal inhibition on feeding by putting LPBN^ObR-hM4di^ mice on a fast-refeeding assay. CNO (1 mg/kg) caused no changes in either food intake or body weight in LPBN^ObR-hM4di^ mice compared to saline controls (Figures 3C and D). These results suggest that inhibition of LPBN^ObR^ neurons does not affect feeding. We next tested to see if LPBN^ObR^ neurons could regulate either glucose tolerance or insulin sensitivity. To this end, we tested LPBN^ObR-hM4di^ mice on a glucose-tolerance test and an insulin-tolerance test. CNO injections caused no changes in either of the tests (Figures 3E and F), suggesting that inhibition of LPBN^ObR^ neurons does not affect blood glucose levels in either context. Taken together, these results suggest that LPBN^ObR^ neurons may not be necessary in affecting glucose homeostasis.

## Discussion

In this study, we sought to identify the electrophysiological effects of leptin on the LPBN and to identify its functional significance. We found that activation of ObR in the LPBN caused inhibition of these neurons at both the cellular level and at the synaptic level. We speculate that the increased potassium conductance that leptin exerts on LPBN^ObR^ neurons may potentially be due to the opening of the ATP-sensitive potassium (K_ATP_) channels, which have been shown to mediate leptin-induced hyperpolarisation in the ventromedial hypothalamus (Spanswick et al. 1997) and the nucleus tractus solitarii (Williams and Smith 2006).

However, mimicking this inhibition *in vivo* caused no changes in the metabolic parameters tested. These results are surprising given that a separate study showed that activation of LPBN^ObR^ neurons is capable of enhancing the counter-regulatory response (Flak et al. 2014). In our study, we found that chemogenetic inhibition of LPBN^ObR^ neurons had no effect on blood glucose whereas it was previously reported that this manipulation resulted in decreased blood glucose levels. We suspect the discrepancy in results might have occurred due to methodological differences. 2-deoxy glucose was used in those studies whereas our study was restricted to the use of insulin and glucose. Insulin might be expected to activate insulin receptors in other parts of the body and brain which may influence the results; and 2-deoxy glucose and glucose may have different mechanisms of action in raising blood glucose levels. We also note that sample sizes used in Flak *et al* were larger than those used in our study (Flak et al. 2014), which raises the possibility that we may not have detected any differences simply due to our lower sample size and thus lower statistical power. Nevertheless, our electrophysiological results are consistent with those reported in Flak et al. (2014) in that leptin serves to inhibit LPBN neurons. Past studies have shown that leptin infusions into the LPBN are able to decrease food intake in rats (Alhadeff et al. 2014), which is also not consistent with what we found. These differences may be due to differences in species: leptin infusion into rats causes induces c-Fos expression in the LPBN (Elmquist et al. 1997) whereas leptin hyperpolarises LPBN neurons in mice (Flak et al. 2014). Finally, we note that chemogenetic manipulation does not fully mimic the effects of leptin with regards to decreasing mEPSC frequency. This both limits the interpretation of our *in vivo* results, with regards to the *in vivo* function of leptin in the LPBN and may also partly account for why we failed to see any phenotype.

Another remaining question from our study is the source of leptin-sensitive mEPSCs to LPBN^ObR^ neurons. One possibility is that these originate from the nucleus tractus solitarii, which contain glutamatergic neurons (Kang et al. 2007) and have been shown to be inhibited by leptin, also through an increased potassium conductance (Williams and Smith 2006).

In conclusion, we found that leptin inhibits LPBN neurons while not affecting feeding or glucose homeostasis. This study raises the possibility that leptin has an unidentified function that is not related to feeding or glucose metabolism at the LPBN.

## Author contributions

K.W.W and J.-W.S conceptualised the study. S.P, K.W.W and J.-W.S designed the experiments. S.P conducted experiments and analysed data. S.P and J.-W.S wrote the manuscript.
